# Combined intermittent hypoxia and surface muscle electrostimulation as a method to increase peripheral blood progenitor cell concentration

**DOI:** 10.1186/1479-5876-7-91

**Published:** 2009-10-29

**Authors:** Ginés Viscor, Casimiro Javierre, Teresa Pagès, Josep-Lluis Ventura, Antoni Ricart, Gregorio Martin-Henao, Carmen Azqueta, Ramon Segura

**Affiliations:** 1 Departament de Fisiologia - Biologia, Universitat de Barcelona, Av. Diagonal, 645 E-08028 Barcelona, Spain; 2 Departament de Ciències Fisiologiques II, Universitat de Barcelona, Feixa Llarga s/n, L'Hospitalet de Llobregat, Barcelona, Spain; 3 Hospital Universitari de Bellvitge, Feixa Llarga s/n, L'Hospitalet de Llobregat, Barcelona, Spain; 4 Centre de Transfusió i Banc de Teixits (CTBT), Unitat de Teràpia Cellular, Feixa Llarga s/n, L'Hospitalet de Llobregat, Barcelona, Spain

## Abstract

**Background:**

Our goal was to determine whether short-term intermittent hypoxia exposure, at a level well tolerated by healthy humans and previously shown by our group to increase EPO and erythropoiesis, could mobilize hematopoietic stem cells (HSC) and increase their presence in peripheral circulation.

**Methods:**

Four healthy male subjects were subjected to three different protocols: one with only a hypoxic stimulus (OH), another with a hypoxic stimulus plus muscle electrostimulation (HME) and the third with only muscle electrostimulation (OME). Intermittent hypobaric hypoxia exposure consisted of only three sessions of three hours at barometric pressure 540 hPa (equivalent to an altitude of 5000 m) for three consecutive days, whereas muscular electrostimulation was performed in two separate periods of 25 min in each session. Blood samples were obtained from an antecubital vein on three consecutive days immediately before the experiment and 24 h, 48 h, 4 days and 7 days after the last day of hypoxic exposure.

**Results:**

There was a clear increase in the number of circulating CD34+ cells after combined hypobaric hypoxia and muscular electrostimulation. This response was not observed after the isolated application of the same stimuli.

**Conclusion:**

Our results open a new application field for hypobaric systems as a way to increase efficiency in peripheral HSC collection.

## Background

Stem cells (SCs) are primitive cells with the potential to differentiate into mature cells [1]. An increase in SCs is observed after various events such as myocardial infarction [2], dilated myocardiopathy [3], cardiac surgery with cardiopulmonary bypass [4], twelve weeks of physical exercise [5,6], menstruation [7], cessation of smoking [8], and in animals or human cells subjected to deep hypoxia conditions in vitro [9-12].

Several studies have found that elevated concentrations of SCs correlate with better clinical outcomes [13], since they possess a general regenerative capacity in blood vessel disorders [14]. Various methods of SC delivery have been shown to be beneficial, mostly with autologous bone marrow cell transplantation [15-17]. No significant differences were found when bone marrow cells or SCs from peripheral blood were compared [18], nor when the comparison was made between bone marrow cells and adipose tissue-derived SCs [19].

An EPO-induced increase of hematopoietic stem cells (HSCs) has been detected in healthy individuals and in patients with renal anemia at two weeks post-administration [20]. Moreover, an EPO-induced mobilization and homing of HSCs and their mediated neovascularization has also been reported in rats after post-myocardial infarction heart failure after six weeks of treatment [21].

Historically, intermittent hypoxia exposure sessions have been used to improve the physical condition and to treat several illnesses, mostly in the countries of the former Soviet Union, although this has been done without a clear understanding of their holistic effects [22]. At all events, this practice has now become widespread in the sport world, and there are even several commercialized forms. Hypoxia exposure has been combined with normal athletic training according to different patterns [23], the most widely-adopted at present being the living-high training-low model [24].

The different forms of standard physical exercise can be difficult to apply with hypoxic procedures, especially in some patients with severe obesity, osteoarticular conditions, neurological sequelae, etc. In contrast, muscle electrostimulation can be easier to apply and has been shown to be as efficient in mimetizing training effects [25-27]. However, intermittent hypobaric hypoxia exposure has been demonstrated to be an efficient stimulus for eliciting adaptive responses in myocardium [28] and skeletal muscle [29].

The aim of the present study was to determine whether it was possible to increase blood SC concentration by means of: 1) short-term intermittent hypoxia, at levels well tolerated by healthy humans and previously demonstrated by our group as being capable of increasing EPO and stimulate erythropoiesis [30] and 2) muscular electrostimulation alone or combined with the aforementioned hypoxia.

## Methods

### Subjects and procedures

Subjects were four healthy males, all members of the research group (AR, CJ, GV and JLV), without toxic habits or medication and with different levels of habitual physical activity (one jogger 4 days/week, one gym user, also 4 days/week, and two without regular physical training). Their mean age was 54.3 (range 46-60), mean height 175 cm (range 170-182), and mean body mass 85.5 kg (range 75-89). They were each subjected to three different protocols: one with only a hypoxic stimulus (OH), another with a hypoxic stimulus plus muscle electrostimulation (HME) and the third with only muscle electrostimulation (OME) [see additional file 1]. In order to avoid undesired interactions, each experimental set was performed at least three months after the preceding one. A hypobaric hypoxia stimulus was applied in a computer-controlled hypobaric chamber [see additional file 2] (CHEx-1; Moelco, Spain) for 3 h on three consecutive days, always from 5 to 8 a.m. (subjects having spent the previous week following the habitual diet and physical activity and with no detected illnesses or chronobiologic changes); the simulated altitude was 5000 m (400 mmHg = 533 hPa), reached in 10 min and returning to sea level pressure in 15 min.

Muscle electrostimulation was applied by means of a Winform Stimulation System (Model W5 multi frequency training, Winform S.r.l., Venice, Italy) according to a widely accepted procedure and following previously described general characteristics [31]. Surface electrodes were fixed on both knee extensors and abdominal wall muscles. Stimulation was achieved at the maximal tolerated intensity (regulated individually by each experimental subject) during two periods of 25 min, one in the first-half period of hypobaric chamber stay (90 min) and the other in the second 90-min period of stay. The protocol of OME was the same as HME and also took place into the hypobaric chamber; however, as the door was open there was no hypoxic stimulus. Oxygen arterial saturation was measured at rest during each hypoxia exposure session by means of a pulsioxymeter (Onyx II 9550, Nonin Medical Inc., Plymouth, MN). The study was conducted according to the Helsinki Declaration and the experimental protocol was approved by the institutional ethics committee.

### Blood sampling, CD34 staining and flow cytometry assay

In order to detect possible individual oscillations, baseline blood samples were drawn on each of the three days prior to the first experiment (OH). Subsequently, blood samples were always obtained just before each of the experimental sets (OH, HME and OME) and 24 h, 48 h, 4 and 7 days later. In the third protocol (OME) an additional sample was taken 10 days after the end of muscular electrostimulation. All samples were obtained between 6 and 8 a.m. following the same extraction methodology as detailed below. Samples were preserved, without any previous processing, at a temperature between 4 and 6°C until transfer to the hematology laboratory. There they were processed according to a blinded design (the technicians involved had no knowledge of either the experimental subject or the protocol).

Peripheral blood samples were collected by puncture of an antecubital vein and placed in tubes treated with 0.34 M di-potassium ethylenediaminetetraacetic acid anticoagulant. All samples were stored at a temperature of 4°C and processed within 24 h of arrival at the laboratory. Blood cell count was assessed by use of an automatic cell counter (AcT-diff; Beckman Coulter, Miami, FL). Samples were incubated for cytometric absolute count with anti-human fluorescein isothiocyanate (FITC)-conjugated CD45 monoclonal antibody (Beckman Coulter, clone J.33) and anti-human phycoerythrin (PE)-conjugated anti-CD34 (clone 8G12, Becton Dickinson) in PBS containing 1% albumin and 0.1% sodium azide for 15 min at room temperature. Red blood cells were lysed with 1 ml of quick lysis solution (CYT-QL-1, Cytognos) for 15 min at room temperature. Samples were incubated under dark conditions and analyzed immediately. To ensure accuracy, reverse pipetting was used to dispense the volumes.

A single-platform protocol with Perfect-Count microspheres CYT-PCM-50 (Cytognos, Salamanca, Spain) was used according to manufacturer's instructions. The Perfect-Count microspheres system contains two different fluorospheres in a known proportion (A and B beads), thus assuring the accuracy of the assay by verifying the proportion of both types of beads. Known volumes (25 μl) of Perfect-Count Microspheres were added to the same known volume (25 μl) of stained blood in a lyse-no-wash technique, and the beads were counted along with the cells. Cell viability was measured by staining the samples with the vital dye 7-aminoactinomycin D (7-AAD), as proposed by the ISHAGE guidelines [32]. Samples were analyzed on a FACScan Scalibur flow cytometer (BD Biosciences) with a 488-nm argon laser and Cell Quest 3.1 software (BD Biosciences). The instrument was aligned and calibrated daily using a three-color mixture of Calibrite™ beads (BD Biosciences) with FACSComp software (BD Biosciences). The gating strategy followed also ISHAGE guidelines [32].

### Statistical analyses

The non-parametric Friedman test for repeated measures was used. All tests were performed using SPSS v.14. Statistical significance was set at P < 0.05. Values are expressed as the median value ± standard deviation (SD).

## Results

**Figure 1:**
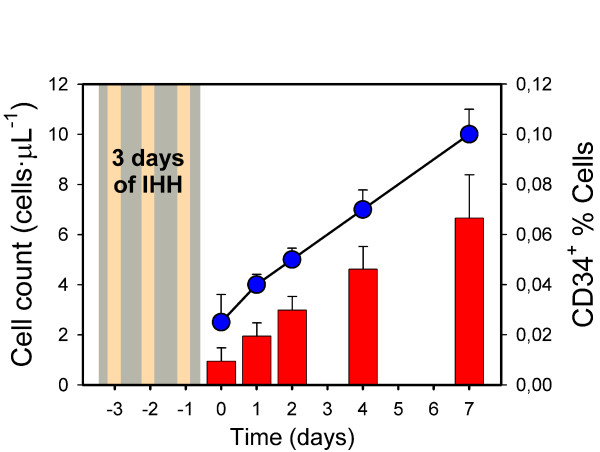
**CD34+ cells after hypobaric hypoxia and muscle electrostimulation**. Evolution of the CD34+ cell count (left axis; red bars) and percentage (right axis; blue circles) during the HME experimental set. Category medians and positive standard errors are shown for the two variables. A statistically significant increase for CD34+ concentration (cells/μL) was found (p = 0.009).

Only the HME experimental data set showed a clear increase for all the subjects (about 3× fold) in the percentage of circulating CD34^+ ^cells, although no significant differences were detected (p = 0.056). However, the number of circulating CD34^+ ^cells increased in this experiment from a median value of 0.95 cells·μL^-1 ^(range: 0.5-2.1) to reach a median level of 6.65 cells·μL^-1 ^(range: 3.7-10.7), this increase being clearly significant (p = 0.009) (Figure 1).

No other studied parameter showed changes in this experimental block. Furthermore, neither OH nor OME experimental data showed statistically significant changes across the study for general leukocyte parameters or circulating CD34^+ ^cells (Table 1).

**Table 1:** Leukocyte parameters in the three experimental sets

		**Before IHH**			**After 3 days of IHH**				
			
**Sampling days**		**-2**	**-1**	**0**	**1**	**2**	**4**	**7**	**10**
							
*Total leukocyte count*	**OH**	6.4	7.2	7.1	6.7	7.6	7.1	6.6	
		1.10	1.25	1.51	1.53	1.72	1.30	1.32	
	**HME**			7.2	7.0	6.8	6.7	6.9	
				1.93	2.36	0.54	1.33	1.31	
	**OME**			6.2	6.9	7.6	6.9	8.7	7.8
				2.86	0.45	0.71	1.86	1.25	1.17
									
*% Lymphocytes*	**OH**	31.9	31.6	29.4	28.3	30.0	29.2	33.3	
		5.00	4.88	6.68	7.01	6.65	6.88	6.03	
	**HME**			40.0	27.9	32.4	47.1	35.7	
				7.50	7.29	5.39	10.53	6.24	
	**OME**			43.2	36.2	31.7	27.5	31.7	34.7
				5.40	2.80	5.61	7.14	8.88	6.14
									
*% MNC*	**OH**	43.2	44.2	40.4	37.4	41.0	41.0	42.3	
		3.80	3.04	7.08	7.78	8.53	7.75	6.44	
	**HME**			40.5	30.0	42.9	33.7	30.6	
				7.67	8.74	7.46	7.18	6.06	
	**OME**			42.7	38.2	34.3	41.6	44.1	43.3
				4.20	3.02	5.31	7.40	10.03	6.71
									
*% CD34*^+^	**OH**	0.081	0.050	0.064	0.063	0.061	0.050	0.075	
		0.006	0.040	0.014	0.017	0.012	0.026	0.023	
	**HME**			0.025	0.040	0.050	0.070	0.100	
				0.017	0.008	0.019	0.036	0.030	
	**OME**			0.050	0.040	0.055	0.035	0.045	0.045
				0.013	0.010	0.017	0.019	0.024	0.028
									
*CD34*^+^*/μL*	**OH**	4.60	3.20	4.55	4.04	4.20	3.95	5.35	
		0.81	3.36	1.92	1.18	1.14	2.09	1.93	
	**HME**			0.95	1.95	2.99	4.62	6.66	
				0.71	0.71	1.36	2.61	2.91	
	**OME**			3.30	2.30	3.45	2.30	3.80	3.60
				1.06	0.92	1.46	2.42	2.09	2.53

Data are median values and standard deviations. Total leukocyte count and subtype percentages were assessed by automatic cell counter. CD34+ absolute concentration (cells/μL) and percentage were obtained by flow cytometry.

## Discussion

The main result of the present study is the synergic capacity of a short-term intermittent hypoxic stimulus plus surface-electrode muscle electrostimulation to increase the circulating concentrations of hematopoietic CD34^+ ^stem cells in a group of four healthy men aged around 50 years old. This increase can be considered as substantial, because it is generally accepted that a concentration of 7 cells/μL is equivalent to approximately 5·10^5 ^cells·kg^-1 ^in an adult subject. This concentration can be assumed to be useful for harvesting purposes and corresponds to a considerable fraction of the increase in CD34^+ ^cells obtained after a standard five-day treatment involving two-day doses of G-CSF (personal data).

It also seems that the increases in CD34^+ ^produced by G-CSF have a non-progressive tendency, as reported in a study of patients with myocardial infarction, in whom circulating CD34^+ ^levels began to decrease the day after the fourth consecutive dose of G-CSF, reaching the previous concentrations between days 6 and 10 after the end of G-CSF treatment [33]. In the present study, CD34^+ ^levels appear to continue increasing 7 days after the last hypoxia session, and thus it is not clear if a plateau or maximum value has been reached. It should also be taken into account that G-CSF shows some pro-thrombotic effects[34,35].

The lack of response in the OHE experiment does not seem attributable to the age of the study participants, since a clear HSC response to physical exercise was detected in a group of 63-year-old men [6]. However, there are alternative explanations for these findings: 1) the relatively short duration of the hypoxic stimulus (a total of 9 h), whereas positive neurogenesis in rats was demonstrated after applying a hypoxic stimulus of 4 h per day over two weeks [9], while other studies detected a positive SC response to physical exercise after about three months of routine physical activity [5,6]; at all events 7 days are enough after myocardial infarction to increase the number of CD34^+ ^cells [36] and a single intense exercise test is able to increase HSC 24-48 h after an exercise bout [37,38]; or 2) the low intensity of the stimulus in our study (used in order to be applied and tolerable to a large majority of healthy people) compared with some in vitro studies, in which clearly more hypoxic atmospheres were used [10,11]. Obviously, a higher number of repeated hypoxia sessions could be applied; however, it does not seem reasonable to use much more intense (higher simulated altitude) or longer hypoxic sessions as these might not be tolerated by some people or patients.

It is also worth noting some of the advantages of muscular electrostimulation over exercise during hypoxia exposure: a) it is easy to measure and reproduce; b) it can be applied in a hypoxic atmosphere (hypobaric chamber or breathing a hypoxic mixture); and c) it can be applied to the majority of humans, even those with mild or severe physical limitations for standard exercise. It is not clear from the present study whether muscular electrostimulation should necessarily be applied simultaneously during hypoxia exposure.

The major limitations of the present study are the short total duration of the hypoxic stimulus in OHE (which was sufficient in HME) and the small sample size; however, given the results it does not seem very likely that a larger sample size would produce significant differences. The lack of a more complete hematologic study means we cannot rule out the possibility that the CD34^+ ^increase is caused by a decrease in "homing" mechanisms in possible target tissues, although this does not seem a likely phenomenon in this case.

Regrettably, our protocol is unable to determine the optimal stimulation timing in order to produce a stable increase in CD34^+ ^cells, although the apparent maintained effect observed (CD34^+ ^increasing 7 days after the stimulus) suggests that some repeated "doses" might alone be enough.

Further studies are required to address several questions derived from the present research: a) the potential repercussions of the detected CD34^+ ^increase on different pathologies, it perhaps being possible to increase HSC homing in injured tissues because after the release of HSCs from bone marrow, cells home to ischemic or damaged regions via alterations of the affected tissue [39]; b) determining the most efficient protocols to induce an optimal and maintained increase in HSC; c) the possibility that the OH or OME stimulus applied via more persistent schedules might also induce a measurable increase in HSC; and d) the need for a more exhaustive study of the possible subclasses of SC released under HME conditions.

## Conclusion

1) A simple protocol stimulating healthy humans with hypoxia plus muscle electrostimulation can quickly induce a notable increase in blood HSC.

2) The significant differences obtained in the HME experimental set over such a short period of time, coupled with the easy application of these two combined stimuli, make this method an interesting tool to increase efficiency in peripheral HSC collection.

## Competing interests

This study has been performed without support form any public or private fund, agency or company. The authors declare that they have no competing interests.

## Authors' contributions

GV: conception and design of the study, experimental subject, collection and/or assembly of data, data analysis and interpretation, manuscript writing, final approval of manuscript; CJ: conception and design of the study, experimental subject, collection and/or assembly of data, data analysis and interpretation, manuscript writing; TP: conception and design of the study, collection and/or assembly of data, data analysis and interpretation, manuscript writing; JLV: conception and design of the study, experimental subject, collection and/or assembly of data, data analysis and interpretation, manuscript writing; AR: conception and design of the study, experimental subject, collection and/or assembly of data, data analysis and interpretation, manuscript writing; GMH: collection and/or assembly of data, data analysis and interpretation, manuscript writing; CA: collection and/or assembly of data, data analysis and interpretation, manuscript writing; RS: data analysis and interpretation, manuscript writing. All authors read and approved the final manuscript.

## Supplementary Material

Additional file 1 **GV and CJ during HME protocol**. The intensity of muscle electrostimulation can be observed in this short movie. Click here for file

Additional file 2 **CHEx-1 Hypobaric chamber**. The hypobaric chamber into BioPol facility at University of Barcelona Campus Bellvitge. Click here for file
