# Supplementary figures and images for: Combined intermittent hypoxia and surface muscle electrostimulation as a method to increase peripheral blood progenitor cell concentration
Source: J Transl Med. 2009 Oct 29;7:91. doi: 10.1186/1479-5876-7-91 (PMC2774674; doi:10.1186/1479-5876-7-91)

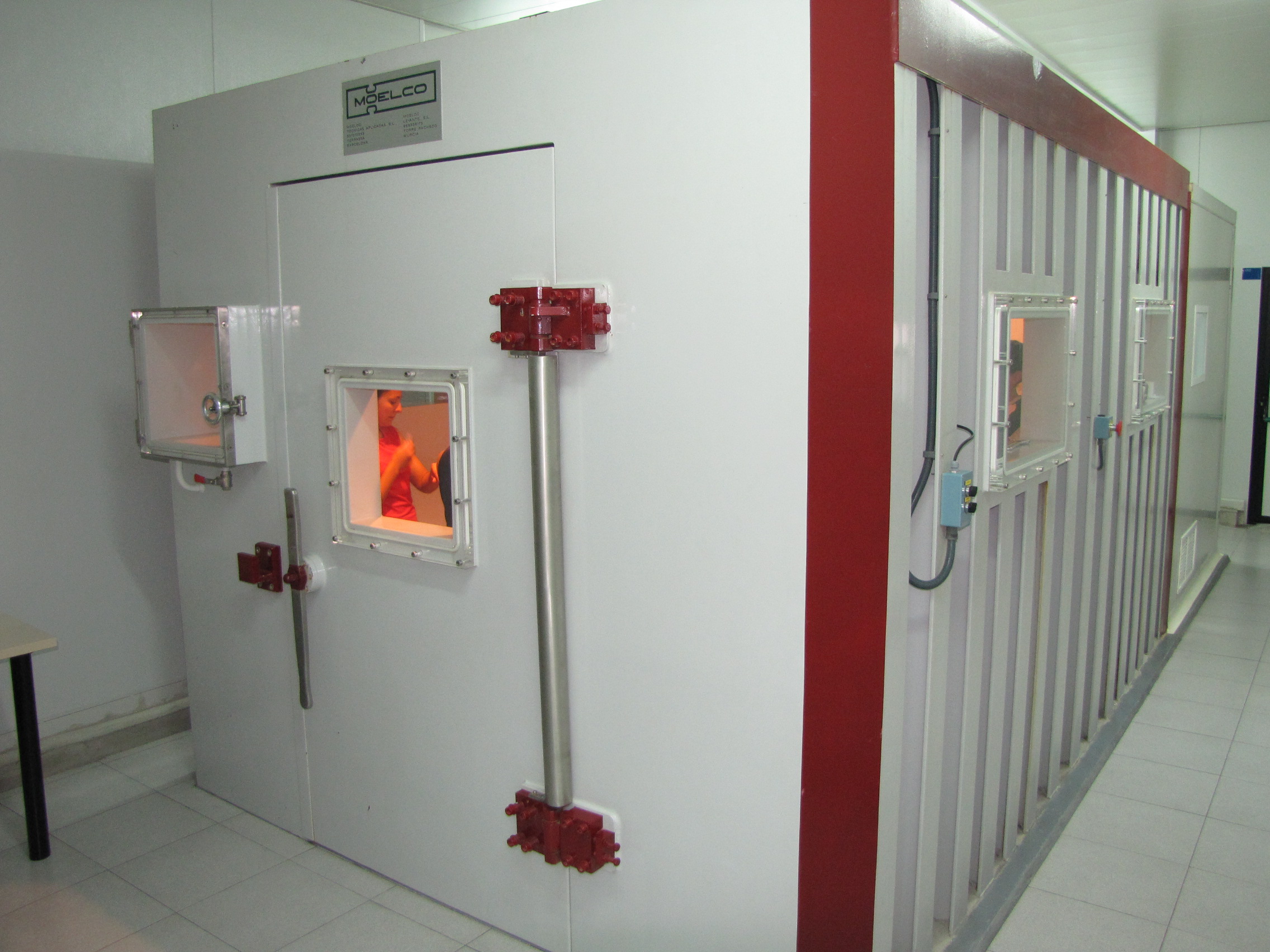

Supplement: Additional file 2 — CHEx-1 Hypobaric chamber. The hypobaric chamber into BioPol facility at University of Barcelona Campus Bellvitge. [file 1479-5876-7-91-S2.jpeg]
